# Editorial: Exercise physiology and gastrointestinal disorders

**DOI:** 10.3389/fspor.2024.1404388

**Published:** 2024-04-10

**Authors:** Moisés Tolentino Bento da Silva, Daniel Moreira-Gonçalves, Francisco Leonardo Torres-Leal, Arménio Aguiar dos Santos

**Affiliations:** ^1^ Laboratory of Physiology, Department of Immuno-Physiology and Pharmacology, Institute of the Biomedical Science Abel Salazar – ICBAS, School of Medicine and Center for Drug Discovery and Innovative Medicines (MedInUP) University of Porto, Porto, Portugal; ^2^ Laboratory of Exercise and Gastrointestinal Tract, Department of Physical Education, Federal University of Piaui, Teresina, Brazil; ^3^ Research Center in Physical Activity, Health and Leisure, Faculty of Sport, University of Porto, Porto, Portugal; ^4^ Laboratory for Integrative and Translational Research in Population Health, Porto, Portugal; ^5^ Department of Biophysical and Physiology, Federal University of Piaui, Teresina, Brazil; ^6^ Department of Physiology and Pharmacology, School of Medicine, Federal University of Ceará, Fortaleza, Brazil

**Keywords:** exercise, gut, gastroinestinal symptoms, gastroesophageal reflux, exercise physiology

**Editorial on the Research Topic** Exercise physiology and gastrointestinal disorders

## Introduction

The gastrointestinal tract (GIT) and exercise constitute an important field of research. While moderate exercise has traditionally been considered beneficial for GIT health, athletes are more prone to experiencing dysbiosis and detrimental GIT symptoms such as bloating, fullness, and diarrhea. These symptoms can lead to an “exercise-induced GIT syndrome,” as previously discussed. Several factors contribute to this syndrome, including the ischemia/reperfusion cycle, hypoxia, heat stress, the inflammatory response induced by skeletal muscle, antibiotic use, training characteristics, and dietary patterns.

The upper GIT also experiences repercussions from vigorous exercise, affecting gastric emptying and leading to symptoms such as nausea, bloating, fullness, and vomiting. Therefore, GIT adaptation is crucial for optimizing sports performance, as the stomach and intestines are where carbohydrate and fluid digestion and absorption occur, supplying active muscles. However, there remains a limited understanding of the interactions between moderate exercise and pathophysiological diseases.

**Figure 1:**
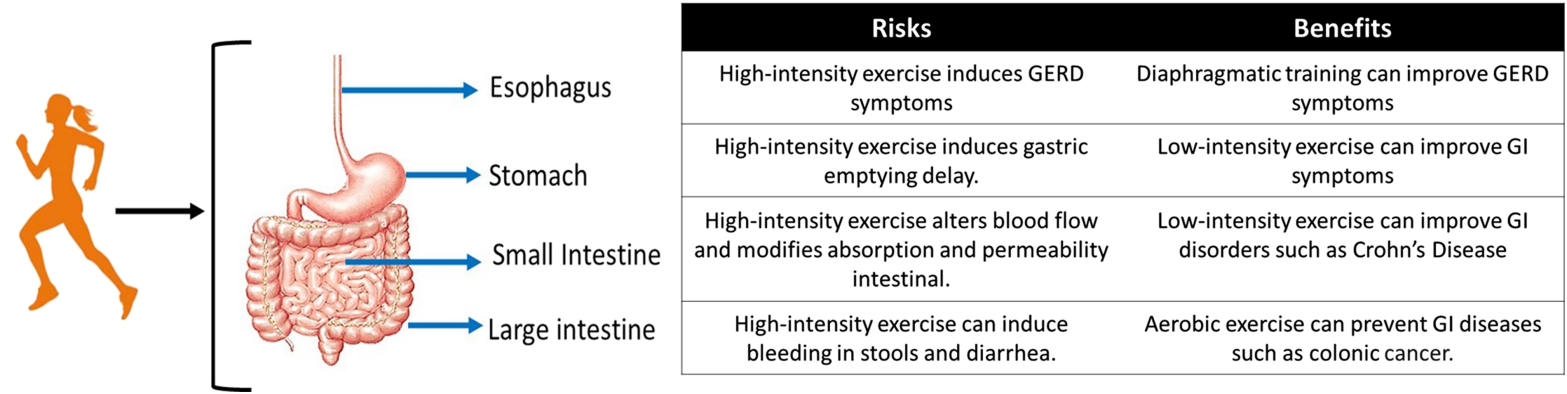
Risks and benefits of physical exercise in different segments of the gastrointestinal tract.

Many studies have investigated the interaction between exercise and gastrointestinal pathophysiological conditions in diseases such as hypertension or dysmotility after cisplatin treatment (1, 2). In this regard, exercise can be utilized as a nonpharmacological treatment. In Figure 1, we have provided a summary of the risks and benefits of exercise in various parts of the GI tract.

The gastrointestinal tract, particularly the intestinal microbiota, provides an extensive and favorable environment for hosted microorganisms that can directly impact various nutritional parameters, including energy expenditure, intestinal nutrient absorption, immunological function, and intestinal barrier function. Additionally, this effect plays a modulatory role in intestinal inflammation through an imbalance in oxidative stress. Certain situations, such as physical exercise, can modify the gastrointestinal microbiome, leading to improvements in metabolic alterations (3).

High-intensity exercise can contribute to various gastrointestinal disorders, which may arise from intestinal inflammation, changes in immunological function, induction of oxidative stress, alterations in nutrition, insufficient recovery periods, and psychological disorders that directly influence the health and performance of athletes (4). During vigorous and prolonged exercise (such as marathons, triathlons, and high-endurance activities), numerous symptoms are described, including abdominal cramps, gastroesophageal reflux disease (GERD), abdominal pain, bleeding in the stool, heartburn, vomiting, urgency to defecate, and a decrease in splanchnic blood flow. The topic aims to identify studies investigating exercise physiology and gastrointestinal disorders. In this research topic, we received eight articles, four of which were accepted and four were rejected.

In the study conducted by Qian et al., the authors explored the association of physical exercise and the gastrointestinal microbiota, as well as their interrelation with possible causality in obese individuals. They observed a great interrelation of obesity and the modifications in the intestinal microbiota using a two-sample Mendelian randomization method. In the obese sample, they noted a reduction in various microorganisms in the microbiome, such as Akkermansia and Intestinimonas. Conversely, there were increases in microorganisms in the microbiome, such as RuminococcaceaeUCG011 and Holdemania. However, these authors suggest that future investigations are necessary to clarify the underlying mechanisms.

In another study (Kelly et al.), the authors investigated the effect of marathon running on the different risk factors associated with gastrointestinal injury and how these injuries can influence gastrointestinal disorders during and after endurance exercise. The study aimed to (a) examine the damage in intestinal cells on marathon day and the post-race day; (b) evaluate the prevalence and gravity of the perception of gastrointestinal disorder symptoms on marathon day and the day after the race, and (c) explore the correlation between risk factors associated with intestinal cell damage and gastrointestinal symptoms. These authors concluded that the individualized nature of gastrointestinal disturbances is associated with gastrointestinal injury and perception of gastrointestinal symptoms in the moment of the exercise as well as post-exercise.

In the systematic review conducted by Cullen et al., the authors assess the role of the different types of exercises on a variety of gastrointestinal microbiomes, diversity in taxonomic composition, and metabolites in the microbial community in humans and animals. They identified 4,731 studies on the topic. After the inclusion and exclusion analysis, the study was narrowed down to 32 papers. These studies observed different exercise protocols, such as aerobic exercise, high-intensity interval training (HIIT), resistance exercise, and a combination of aerobic and anaerobic exercise.

The authors of the study conclude that physical exercise induces a positive impact on the gastrointestinal microbiome. However, they also acknowledge the limitations of the study, particularly regarding the significant variability in exercise protocols used in both humans and animals. This variability poses challenges in standardizing protocols and determining which methodologies are most suitable for future studies. A differentiated understanding of the relationship between physical exercise and the microbiota will be useful for the development of individualized exercise programs to optimize health.

In conclusion, the study of gastrointestinal disorders associated with exercise is an important area of research. While many studies focus on aerobic situations and acute exercise effects, there is a scarcity of research investigating the cellular and molecular mechanisms involved. This body of research is crucial for enhancing our understanding of the behavior of gastrointestinal disorders in physical exercise.
